# Netted crop covers reduce honeybee foraging activity and colony strength in a mass flowering crop

**DOI:** 10.1002/ece3.5154

**Published:** 2019-04-29

**Authors:** Lisa J. Evans, Brian T. Cutting, Mateusz Jochym, Milena A. Janke, Crystal Felman, Sarah Cross, Marine Jacob, Mark Goodwin

**Affiliations:** ^1^ Plant & Food Research Australia c/o Queensland University of Technology Brisbane Queensland Australia; ^2^ The New Zealand Institute for Plant & Food Research Limited Hamilton New Zealand; ^3^ Agrocampus Ouest Rennes France

**Keywords:** colony health, enclosure, foraging behavior, Apis mellifera, pollination, protected cropping

## Abstract

The widespread use of protective covers in horticulture represents a novel landscape‐level change, presenting the challenges for crop pollination. Honeybees (*Apis mellifera* L) are pollinators of many crops, but their behavior can be affected by conditions under covers. To determine how netting crop covers can affect honeybee foraging dynamics, colony health, and pollination services, we assessed the performance of 52 nucleus honeybee colonies in five covered and six uncovered kiwifruit orchards. Colony strength was estimated pre‐ and postintroduction, and the foraging of individual bees (including pollen, nectar, and naïve foragers) was monitored in a subset of the hives fitted with RFID readers. Simultaneously, we evaluated pollination effectiveness by measuring flower visitation rates and the number of seeds produced after single honeybee visits. Honeybee colonies under cover exhibited both an acute loss of foragers and changes in the behavior of successful foragers. Under cover, bees were roughly three times less likely to return after their first trip outside the hive. Consequently, the number of adult bees in hives declined at a faster rate in these orchards, with colonies losing on average 1,057 ± 274 of their bees in under two weeks. Bees that did forage under cover completed fewer trips provisioning their colony, failing to reenter after a few short‐duration trips. These effects are likely to have implications for colony health and productivity. We also found that bee density (bees/thousand flowers) and visitation rates to flowers were lower under cover; however, we did not detect a resultant change in pollination. Our findings highlight the need for environment‐specific management techniques for pollinators. Improving honeybee orientation under covers and increasing our understanding of the effects of covers on bee nutrition and brood rearing should be primary objectives for maintaining colonies and potentially improving pollination in these systems.

## 1 INTRODUCTION

Global food production relies on managed and unmanaged animal pollinators, with at least 75% of crops benefiting from pollinator visits to flowers (Klein et al., 2007). Land management changes within and surrounding production ecosystems can have a profound effect on pollinator assemblages and their flower‐visiting behavior (Bates et al., 2011; Kremen, Williams, & Thorp, 2002; Stavert, Pattemore, Bartomeus, Gaskett, & Beggs, 2018), with consequences for pollination and pollinator management. The widespread and extensive use of protective covers in horticulture represents a novel landscape‐level change, which is likely to affect pollination systems.

High‐value crops, which were traditionally grown in the open, are increasingly produced under netting and/or plastic covers (Baudoin et al., 2017; Castilla, 2002; Cook & Calvin, 2005; Reddy, 2016). Crop covers are used to increase the reliability or duration of production by modifying the growing environment and to enhance crop quality and yields by providing a physical safeguard against extreme weather, plant pests, and pathogens (Amarante, Steffens, & Argenta, 2011; Lloyd, Hamacek, George, Nissen, & Waite, 2005; Marco et al., 2008; Middleton & McWaters, 1997; Sauphanor, Severac, Maugin, Toubon, & Capowiez, 2012). For crops grown for seed production, covers can prevent foreign pollen contamination, which can cause reduced yields or undesirable hybrids (Morison, Vaissiere, Martin, Pecaut, & Cambon, 2000; Rodet, Torre Grossa, & Bonnet, 1991).

While there are benefits to protected cropping, covers may have unintended negative consequences for certain aspects of production. The physical barrier presented by plastic or small‐aperture mesh can reduce pressure from pests, but also restrict movement of beneficial insects including pollinators. Furthermore, while the environmental conditions created under covers may support plant growth, they may not be favorable for pollination and/or the health of pollinators (Dag, 2008; Free, 1993; Middleton & McWaters, 1997; Pinzauti, 1994).

Honeybees (*Apis mellifera*) are important pollinators in many horticultural environments (McGregor, 1976; Morse & Calderone, 2000), but when located under covers they can be less active or become unevenly distributed in a system (Dag & Eisikowitch, 1995; Leech, 2014; Middleton & McWaters, 1997). Under covers, colony strength has been observed to decline rapidly, possibly because of climatic conditions or reduced access to pollen and nectar resources (Dag, 2008; Free, 1993; Pinzauti, 1994). Despite the importance of honeybees to the horticultural industry, to the best of the authors’ knowledge no empirical study has comprehensively quantified the effects of crop covers on honeybee behavior, colony health, and pollination services. An approach that considers all of these factors simultaneously will enable a better understanding of the degree to which honeybees are affected by crop covers, and the causes of these impacts. This level of understanding is critical for devising mitigation strategies to improve the effectiveness and sustainability of honeybee pollination in these environments.

New Zealand kiwifruit orchards are a useful model environment for studying the effects of crop covers on pollinators and pollination. In New Zealand, around 20% (800 ha) of gold‐fleshed kiwifruit (*Actinidia chinensis* var. *chinensis* “Zesy002”; commonly known as Gold kiwifruit) were covered with hail netting between 2013 and 2015, with more covers being installed each year (Cutting et al., 2018). This variety of kiwifruit is sensitive to wind and hail damage, which causes cosmetic impairment to fruit and increases susceptibility to bacterial infection by *Pseudomonas syringae pv. Actinidiae* (Psa) (Beth Kyd, Zespri Ltd. Pers comm). Covering orchards with netting helps to prevent such damage. However, kiwifruit relies on insect and wind pollination for fruit set (Costa, Testolin, & Vizzotto, 1993; Craig, Stewart, Pomeroy, Heath, & Goodwin, 1988) as it is a dioecious plant; staminate and pistillate flowers are borne on separate vines (typical planting ratio—1:8 staminate:pistillate; Sale, 1981). Wind alone is inadequate for the production of marketable kiwifruit (Burge, Spence, & Pallesen, 1988; Costa et al., 1993; Testolin, Vizzotio, & Costa, 1991; Vaissiere, Rodet, Cousin, Botella, & Torre Grossa, 1996), and the New Zealand industry is heavily dependent on managed honeybee pollination; 92% of insect visitors to kiwifruit flowers are honeybees (Howlett, Read, et al., 2017), although some growers also use “artificial pollination”, spraying or blowing pollen within the orchard. When fully enclosed, the netting covers will greatly reduce the effectiveness of wind pollination (as seen with shelterbelts: Burge et al., 1988) and will prevent the passage of honeybees (and other larger pollinators such as bumblebees) in and out of the crop.

The current study was conducted as a result of increasing concern around the availability and sustainability of honeybees for pollination of covered kiwifruit. In New Zealand, kiwifruit flowering occurs just prior to *Leptospermum scoparium* (mānuka) flowering. If hive quality declines during kiwifruit pollination, it may lower subsequent production of valuable mānuka honey. These concerns have led to increased costs and tight restrictions for hive use in kiwifruit (Beth Kyd, Zespri Ltd. Pers comm). We use an orchard‐level comparison (covered vs. uncovered) to assess the effect of netting covers on honeybee foraging activity, colony strength, and per‐bee pollination efficacy in kiwifruit.

## 2 MATERIALS AND METHODS

### 2.1 Study system

**Figure 1:**
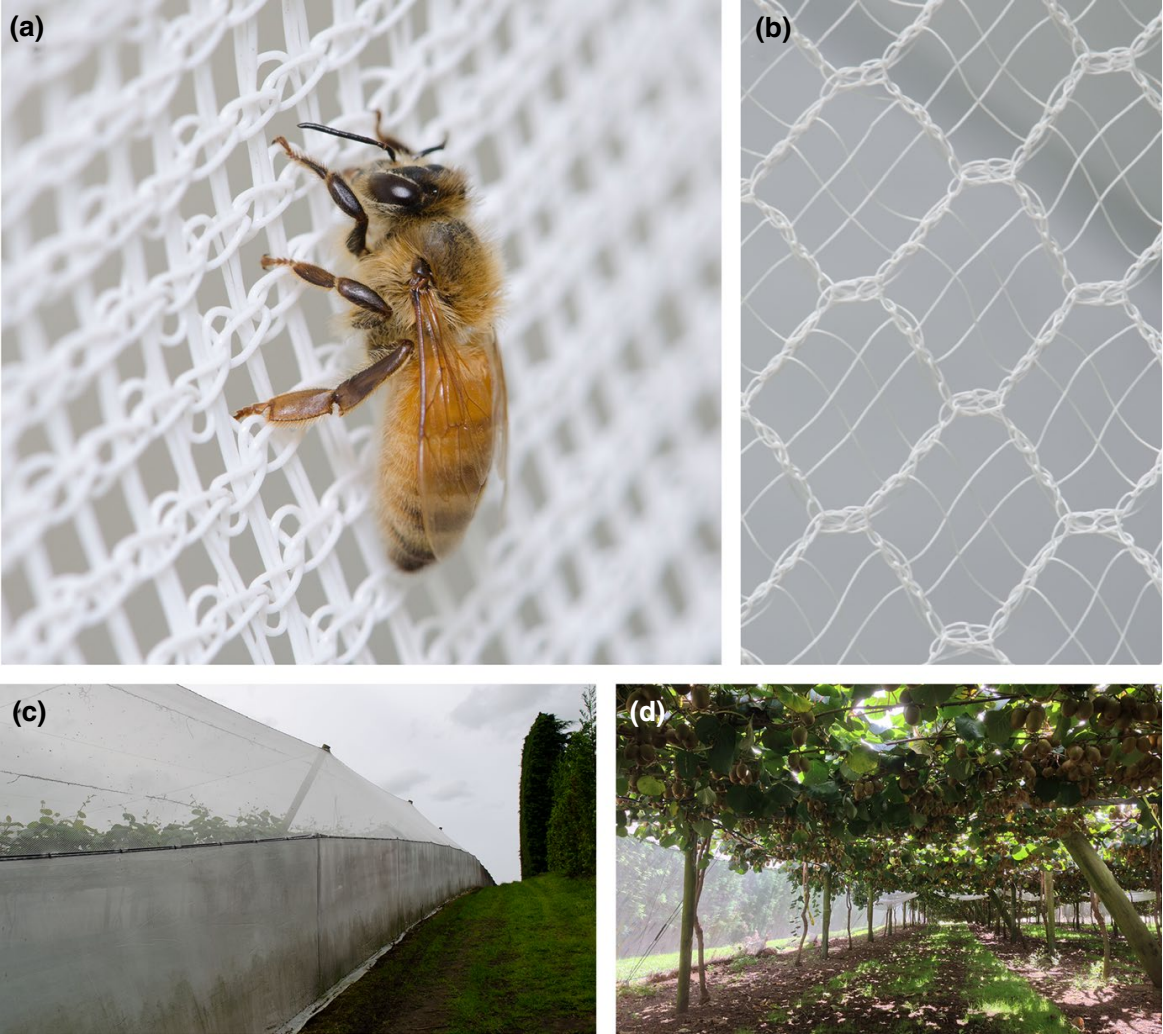
Production of kiwifruit (*Actinidia chinensis*) under netting; honeybees appear to become disorientated and are observed hanging from side netting (a). Hail netting with a maximum gap size of 6mm is used for the canopy of enclosures (b), and a more densely woven netting is frequently used for the sides of the enclosures (c). Kiwifruit vines are grown over pergolas within the enclosure (d)

This study was carried out on five covered and six uncovered (control) kiwifruit orchards located in the Bay of Plenty region of New Zealand. Sampling was undertaken within sections (blocks) ranging between 0.3 and 10.1 ha (mean = 1.8 ha; Table 1). The precise design of the covers differed among orchards; however, they were all completely enclosed with white, fine woven plastic netting (e.g., Figure 1). The recommended honeybee stocking for uncovered kiwifruit is 8–12 hives/ha, depending upon the number of flowers open (Clinch, 1990; Goodwin, 1986; Palmer‐Jones, Clinch, & Briscoe, 1976). In this trial, honeybee stocking rates were controlled by orchard managers and varied between 7 and 10 hives/ha (Table 1). Honeybee abundance in the uncovered orchards may have been influenced by landscape‐level factors outside of our control, including the density of managed honeybees on nearby properties, and availability of competing floral resources.

**Table 1:** Orchard‐specific information for focal and nonfocal orchard blocks

Orchard	Treatment (covered/ uncovered)	Block size (ha)	Grower hives/ha	Additional nucleus hives installed for trial	Supplementary pollination
A (Focal)	Uncovered	0.9	7	8	No
B (Focal)	Covered	10.1	8	8	Yes
C	Covered	2.6	8	4	No
D	Covered	1.3	8	4	No
E	Covered	1.0	10	4	No
F	Covered	0.5	10	4	No
G	Uncovered	0.8	10	4	No
H	Uncovered	0.6	10	4	No
I	Uncovered	0.4	8	4	No
J	Uncovered	0.7	8	4	No
K	Uncovered	0.3	8	4	No

Note

Colony strength (adult bees) was assessed in all additional nucleus hives, deployed across all orchards (A–K). The assessment of individual honeybee foragers and pollination were conducted in focal orchards (A and B).

Intensive monitoring was undertaken in two of these 11 orchards (referred to hereafter as focal orchards): a 10.1 ha covered orchard (netting: white thread shading 12%, Ultraviolet block 20%) and a 0.92 ha uncovered orchard, which were 20 km apart. The paired focal orchards were selected a priori because they had similar management techniques (same orchard manager) including honeybee stocking rates and pest control regimes. In the covered orchard only, managers applied milled pollen to flowers using a quadbike mounted pollen duster (Kiwi Pollen^®^), in alternating rows each day during flowering. This blown pollen was applied with the intention of supplementing honeybee pollination rather than fully pollinating the crop; the applied rate of 75 g/ha is much lower than the recommended rate for achieving full pollination without bees, when pollen is applied dry by broadcast blowing (2–3 kg/ha; J. Hamlyn, Kiwi Pollen Ltd. Pers comm.).

### 2.2 Preparation of nucleus hives and assessment of adult bee numbers

Fifty‐two nucleus honeybee hives (five frames each) were prepared on 5 September 2016. Of these hives, 16 (termed “observation hives”) were fitted with a modified entrance/exit tunnel (580 × 100 mm), which was transparent to allow direct observation of bees, and allowed fitting of two radio frequency identification (RFID) readers (ilD^®^ MAJA) used to record bidirectional movement of tagged bees in the tunnel (Supporting Information Figure S1). These readers channel bees through a narrow space, increasing probability of tag detection but reducing the number of bees that can move in and out of a colony at one time. Congestion in a large colony could affect foraging rates; consequently, it was necessary to use small colonies to reduce congestion at the modified hive entrances.

To standardize hives, a virgin queen was introduced into each hive between 5 and 7 September. Colonies were assessed after four weeks to confirm that their queen had begun to lay eggs and that capped brood was present. Colonies were fed with 50% sugar syrup (v/v) throughout the trial and treated for *Varroa destructor* (one Apivar^®^ strip per nuc), a parasite that can reduce homing ability and foraging trip duration in honeybees (Kralj & Fuchs, 2006). Both feeding and varroa treatment are standard practices for honeybees used for kiwifruit pollination in New Zealand (Goodwin, 1986; Goodwin & Houten, 1991; Goodwin, Houten, & Perry, 1991).

Eight of the observation hives were moved into each of the focal orchards in late spring, on 28 October 2016. The remaining 36 nucleus colonies were deployed across the nine other orchards (nonfocal orchards: four covered, five uncovered) in modified Langstroth hives (two nucleus colonies housed side by side, internal dividers and separate entrances kept the two nucleus colonies independent). This hive arrangement facilitated transport of the colonies, which were deployed when 10%–20% of kiwifruit flower buds had opened, between 29 October and 3 November.

The number of adult bees present in each hive (a measure of colony strength) was estimated by photographing both sides of each frame in the hives and by comparing these images to reference photographs of frames with known numbers of bees. Photographs of hive frames were taken at the time of deployment, or within 24 hr prior to transfer into the orchards and again at the end of flowering (after 9–13 days) to determine whether there had been a change in colony strength (for more details see Supporting Information Section S1).

### 2.3 Tagging different cohorts of bees

All tagged bees were obtained from five large “donor hives” and introduced into the 16 “observation hives” to reduce the chances of any observed differences in behavior being correlated with colony of origin. The donor hives were located approximately 2 km from the 16 observation hives to reduce the likelihood of tagged foragers returning to their original colony. Three different cohorts of bees were fitted with RFID tags: naïve foragers, pollen foragers, and nectar foragers.

Honeybees can begin foraging for their colony when they are between 10 and 39 days of age, and most commonly begin between days 18 and 28 (Winston, 1987). To increase our likelihood of having foraging naïve honeybees, newly emerged bees were fitted with RFID tags daily between 23 and 9 days before kiwifruit flowering. While we cannot ascertain that these bees did not forage prior to being deployed in kiwifruit orchards, their average age when they were first recorded foraging was 18.05 days, which is within the age range in which honeybees typically begin to forage (Winston, 1987).

To obtain foraging naïve honeybees, two frames of capped brood were removed from the donor hives each week and incubated at 34°C in the laboratory. Each morning, 80 newly emerged bees were carefully removed from the surface of the frames with forceps and distributed, five per cage, among 16 queen cages (Ecrotek^®^). The bees were provisioned with a drop of water and approximately 3.25 g of queen candy (icing sugar and honey), which blocked one end of the cage. All bees were returned to the incubator for 24 hr to allow their cuticles to fully sclerotize. The following day, the queen cages were placed into a refrigerator to chill bees to quiescence. A patch (c. 2 mm^2^) of scutal pile was removed with a scalpel to improve tag adhesion. RFID tags (Microsensys GmbH: measuring: 2 × 1.6 × 0.5 mm; mass: 4 mg) were attached with cyanoacrylate adhesive (Loctite^®^ Gel Control). We recorded the tag's unique identification number, the date, and the randomly selected observation hive into which the cage was subsequently transferred. Approximately 60 tagged newly emerged bees were added to each hive.

Nectar and pollen foragers were caught and tagged daily for eight days immediately prior to observation hives being moved into flowering focal orchards. Bees from the donor hives were trained to forage for scented sucrose at feeding stations approximately three meters from their hive. Bees at the feeding station (nectar foragers) and bees returning to their colonies with pollen (pollen foragers) were caught and chilled to quiescence and tagged as described above. Between seven and ten pollen or nectar forager were placed into each cage (separate cages were used for nectar and pollen foragers), and 7 g of queen candy was provided. The bees took between one and three days (varying with temperature and number of workers in colony) to consume the queen candy and integrate into their new colony. This gradual introduction increased the likelihood that the tagged bees would be accepted into their host colony without aggression. Some of the tagged bees died in their cage. These bees were removed, their RFID tags scanned, and their IDs removed from the list of tagged bees per colony. A mean of 80 (range = 76–84) nectar and 62 pollen foragers (range = 50–70) were added to each of the 16 observation hives.

### 2.4 Recording the foraging activity of tagged individuals

On 28 October, colonies with RFID‐tagged bees were blocked before the bees had begun foraging and relocated (c. 100 km) to the two focal orchards. In each focal orchard, four tagged hives were placed on the edge of the orchard and four hives nearer the center, 100–150 m from the edge of the orchard, to account for potential effects of location within the orchard. The colonies were opened and given a 24‐hr acclimation period before the RFID readers were fitted into a subset of the tunnels. Eight pairs of readers were used to capture the foraging activity in eight of the 16 colonies at a time. Four pairs of readers remained on four hives (two covered and two uncovered) for the duration of the 12‐day trial. The other four pairs of readers were shifted between colonies after two to five days. After 11 or 12 days, each focal colony was carefully searched and tagged bees within the colony were scanned and identified to account for any tagged bees that may not have left the hive and therefore never registered on the tunnel readers during the experiment.

### 2.5 Assessing pollination: bee abundance, flower visitation rates, and seed production

Effectiveness of pollen transfer by honeybees was characterized in the focal orchards by measuring abundance of honeybees in the orchard, flower visitation rate, and pollen transfer efficiency. Honeybee abundance was measured by conducting instantaneous counts of bees in ten 3 m × 6 m quadrats across each orchard, at four time periods (0900, 1100, 1300, and 1500 hr) distributed over three days. As flower density varied between the orchards and over time, abundance was adjusted to the number of bees observed per 1,000 flowers. Flower density was 2.5 times higher in the uncovered orchard, with an average of 52 (±4.3) flowers per m^2^ compared with 21 (±1.7) in the covered orchard.

To measure visitation rates, two new groups of focal flowers were filmed over three days in each orchard (1 orchard per day). The focal flowers were between one and two‐day postopening and were selected and tagged the night before filming commenced. Activity on these flowers was recorded between 0930 and 1830 hr.

Pollen transfer efficiency was established by quantifying the number of kiwifruit seeds produced by flowers after a single honeybee visit. Kiwifruit size and weight at harvest are dependent on the number of seeds set through pollination (Hopping, 1976), and the number of seeds produced is related to the number of viable pollen grains deposited on stigmas (Hopping, 1990). Between 2 and 6 November, groups of test flowers (bagged prior to opening to prevent insect visitation) were unbagged and observed until a honeybee visited (*n* = 24 and 23 in uncovered and covered orchards, respectively). The visited flower was then labeled with the duration of the visit and rebagged along with an unvisited (control) flower. Any resulting fruit was harvested when mature (20 and 23 March). Missing fruit was not included in the dataset, as fruit may have been absent for reasons other than pollination failure. Seeds per fruit were counted (see Supporting Information Section S2).

During the experiment, orchard managers blew milled kiwifruit pollen up into the canopy at a low rate (75 g/ha) in the covered focal orchard only. While pollen was not directly blown onto our bagged test flowers, more pollen was available to be moved by bees onto test flowers from surrounding flowers. For this reason, in our analysis we included single visit data collected from two additional orchards using the same methods; a covered orchard, which did not have pollen applied artificially and visited in the same year as the current study (*n* = 19), and an uncovered orchard sampled in 2010 (*n* = 18; Goodwin, Evans, Cross, Janke, & Jacob, 2017).

To assess pollination in the focal orchards, 20 open‐pollinated, one‐day‐old flowers were marked in the middle and edge of the orchard, on three days (120 flowers total per orchard). All flowers were open to visits by bees before and after being marked. Missing fruit was not included in the dataset. The numbers of seeds were recorded for all harvested fruit.

### 2.6 Data analysis

All formal statistical analyses were conducted in R v3.5.0 (R Core Team, 2018). Statistical analyses were performed using generalized linear (mixed effects) models (GLM or GLMM, respectively). The appropriate distribution families were determined using a distribution fit (R package MASS; Venables & Ripley, 2002). Minimal models were identified by step‐wise reduction. Significance of terms was established with likelihood ratio tests (LRT). We validated the final models by inspecting the model outputs and diagnostic plots.

#### 2.6.1 Colony strength

Number of adult bees was analyzed separately for the observation hives and the hives in the nine nonfocal orchards because of the differences in their setup (e.g., observation hives had reduced entrances). In the nonfocal orchards, the four nucleus colonies were cohoused in two standard hive boxes. The numbers of bees per hive for the cohoused colonies were averaged prior to analysis, because there was considerable drifting of bees between directly adjacent colonies. The effect of treatment (covered vs. uncovered), day (continuous variable representing the number of days since introduction of hive into the orchard, scaled to 0–1 range), and their interaction on pretrial and posttrial counts of adult bees in hives was tested with a negative binomial GLMM (R package lme4; Bates, Maechler, Bolker, & Walker, 2015). The models included a random intercept for each colony ID. We evaluated model fit with an overdispersion test (R package blmeco; Korner‐Nievergelt et al., 2015).

#### 2.6.2 Foraging behavior

The number and duration of trips individual bees made away from their colonies in the two focal orchards were calculated using a custom R script (see Supporting Information Section S3 for details). Trip duration was defined as the period between two consecutive detections at the outer RFID reader. Using the density distribution of log10‐transformed trip lengths, we define three distinct types of trips: short trips <6 min, foraging trips, and overnight trips (Supporting Information Figure S2). Only foraging trips were included in our analyses, as the other trips may serve a different purpose. For example, short trips may be defecation or orientation flights, which typically last between 2.5 and 5 min (Degen et al., 2015). Overnight trips were infrequent (5% of trips in the covered orchard only) and could also have been due to RFID tag miss reads. Several studies have used similar minimal cutoffs for foraging trip duration in bumblebees (Evans, Smith, & Raine, 2017; Gill, Ramos‐Rodriguez, & Raine, 2012).

#### 2.6.3 Pollination service delivery

Honeybee abundance (bees per 1,000 flowers) was compared in the covered and uncovered focal orchards using a GLMM with a normal distribution and a log link function. The full model consisted of treatment, consecutive day of the trial (both defined the same way as for the colony strength model), their interaction, and random intercepts for quadrat ID and time of day at which the survey was performed (9:00, 11:00, 13:00, and 15:00; coded as ordered factor). The five highest bee counts (outliers) were removed from the dataset to improve fit. The number of honeybee visits received by flowers in focal orchards was compared using a negative binomial GLM (R package *MASS*; Venables & Ripley, 2002). The visitation data were scaled to exactly 8 hr (the raw footage of flowers was between 6.38 and 8.23 hr long) and rounded to nearest integer to approximate counts. Treatment, position (middle vs. edge of the orchard block), and their interaction were included in the full model. The effect of netting covers on single visit seed counts was tested with a GLM with a normal distribution and a log link function. The full model included: treatment, visit duration, and their interaction. Seeds produced by open‐pollinated flowers were compared in the focal orchards using a negative binomial GLM (Venables & Ripley, 2002). The full model included: treatment, position, and their interaction.

## 3 RESULTS

### 3.1 Effect on colony strength

**Figure 2:**
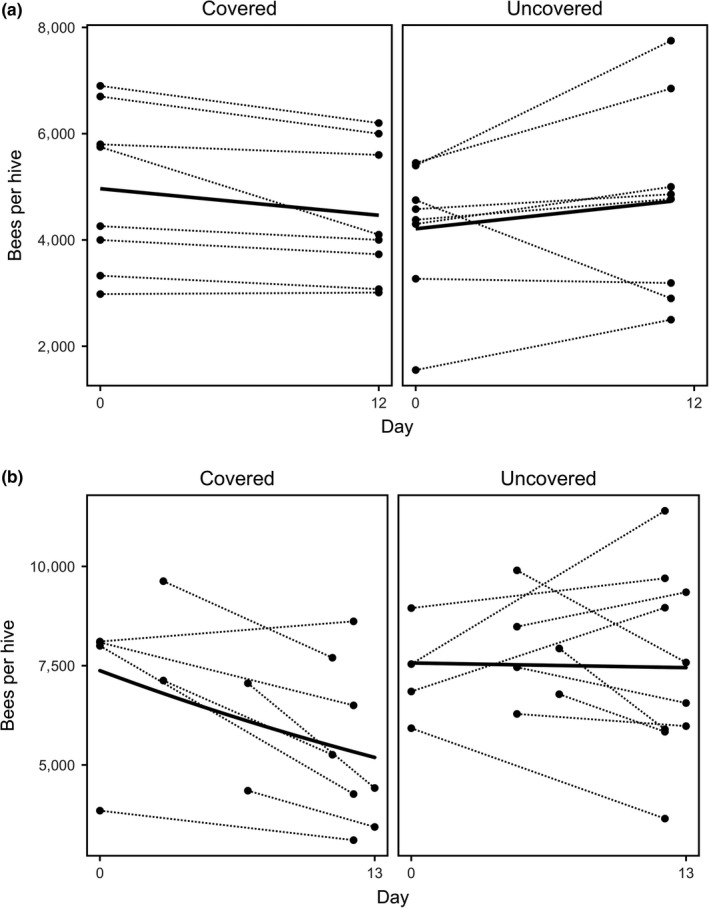
Estimated number of adult honeybees in (a) 16 observation hives at two focal orchards (eight covered and eight uncovered) and (b) 18 hives at nine nonfocal kiwifruit orchards (four covered and five uncovered orchards). In the nonfocal orchards, there were four nucleus colonies were present per site, these were cohoused in two standard hive boxes. The bees per hive for the cohoused colonies were averaged prior to analysis to give ten uncovered and eight covered hives. Honeybee numbers were scored when hives were moved into their respective orchard (day 0–7) and at the end of flowering (day 11–13)

Adult bees in the observation hives were more likely to decline in the covered orchard than in the uncovered orchard between the pre‐ and postassessments, a period of 12 days within the enclosure; *p* = 0.043 (LRT *χ* ^2^ = 4.081; Figure 2a; Supporting Information Table S1 for model summary). Colony strength in the covered orchard declined at an estimated rate equivalent to 20.3 bees per colony per day (mean bees/colony presented in Table 2). In contrast, colony strength in the uncovered orchard increased at an estimated rate equivalent to 22.4 bees per colony per day. A similar pattern was evident across the nine nonfocal orchards (four covered and five uncovered), with adult bee numbers decreasing steeply in the covered orchard (estimated rate equivalent to 87.4 bees per colony per day) and marginally in the uncovered orchards (estimated rate equivalent to 4.7 bees per colony per day). The rate of change was significantly different between the nonfocal covered and uncovered orchards; *p* = 0.030 (LRT; *χ* ^2^ = 4.724; Figure 2b, Supporting Information Table S2 for model summary). Overall, colonies in covered orchards lost an average of 1,057 ± 274 bees, while colonies in uncovered orchards gained an average of 117 ± 422 of bees.

**Table 2:** Change in colony strength (number of adult bees) between the pre‐ and postassessments

Orchard	Treatment	Mean bees/colony when moved into orchard ±*SE*	Mean bees/colony when moved out of orchard ±*SE*
Focal orchards	Covered	4,965 ± 536.4	4,465 ± 455.0
	Uncovered	4,210 ± 450.6	4,728 ± 660.1
Nonfocal orchards	Covered	7,026 ± 697.8	5,413 ± 709.9
	Uncovered	7,611 ± 391.5	7,491 ± 737.7

Note

Preassessments were conducted at the time of deployment, or within 24 hr previous to transfer into the orchards. Postassessments were conducted at the end of flowering, before the hives were removed from the orchard. Hives were in the focal orchards for 11 or 12 days. Hives were in the nonfocal orchards (four covered and five uncovered) for between 9 and 13 days, depending on the duration of flowering.

### 3.2 Effect on foraging activity

RFID data were obtained for 479 of the c. 3,232 tagged bees: 370 in the uncovered focal orchard and 109 in the covered focal orchard. Of these bees, 65% (*n*= 311/479; 275 in the uncovered and 36 in the covered orchard) completed at least one foraging trip ‐ defined as a period away from the hive of > 6 min and < 360 min (Supporting Information Figure S2). Another 13% of bees (*n* = 64/479; 36 in the uncovered and 28 in the covered orchard) only completed trips < 6 minutes, and fewer than 1% the bees (*n* = 3/479; all in the covered orchard) undertook only overnight trips. The remaining 21% of the bees (*n* = 101/479; 56 in the uncovered and 45 in the covered orchard) did not forage for their colony; instead they left their hive and were not detected again.

The likelihood of a bee disappearing outright was close to three times higher for bees under cover, with 41.3% of bees detected (*n* = 25 naïve, 11 nectar, and 9 pollen foragers) failing to return from their first trip outside the hive. This is a conservative estimate of forager loss, as foraging activity was not recorded for all hives every day (eight of 16 colonies were monitored per day), and the RFID readers were not used during the 24‐hr acclimation period on the first day that the hives were in the orchard.

Eighty‐six percent of tagged bees were never detected by a reader (81.5% of tagged bees in the uncovered orchard and 94.5% in the covered orchard). These bees may have never left the hive, or lost their tags, or they were lost or died before readers were fitted to colonies. When colonies were searched at the end of the experiment, 40 of the tagged bees (1.4% of all unrecorded bees) recovered within the colony had never left their colony, and there were no dead tagged bees or free tags within or around the colonies—suggesting that forager loss before reader attachment was the major cause of lack of detection.

### 3.3 Effect on individual foraging behavior

Across the 16 observation hives, 4,220 foraging trips were recorded. The number of foraging trips recorded varied between colonies within an orchard and was positively correlated with the number of foraging bees (bees that completed at least one foraging trip) in each colony (Spearman's ƿ = 0.91 *n* = 14 *p* < 0.001). Bees in the uncovered orchard were more active foragers and continued foraging for a longer period than bees in the covered orchard (Table 3), irrespective of cohort (i.e., naïve, nectar, and pollen foragers; Supporting Information Figure S3). Using only bees from the four colonies that were connected to the RFID system continuously for the duration of the trial, we showed that bees in the uncovered orchard also continued foraging for more days (Table 3). Conversely, the proportion of nonforaging trips made by bees (trips between 0.45 s and 6 min, which we considered to be too short for foraging to occur) was much higher in the covered orchard (48 short trips vs. 57 foraging trips; 45.7%) than in the uncovered orchard (781 short trips vs. 4,163 foraging trips; 15.8%).

**Table 3:** Honeybee foraging behavior in focal uncovered and covered kiwifruit orchards

Foraging behavior	Covered Mean ± *SE*	Uncovered Mean ± *SE*
Foraging trip duration (min)	25.6 ± 7.73	35.0 ± 0.51
Foraging trips per day	1.2 ± 0.08	4.1 ± 0.12
Foraging trips overall	1.5 ± 0.18	15.0 ± 1.05
Number of days foraged	1.2 ± 0.17	5.2 ± 0.34

Note

Data presented were generated using RFID tracking. Means and standard errors are reported for individual honeybee foragers.

### 3.4 Effect on pollination service delivery

**Figure 3:**
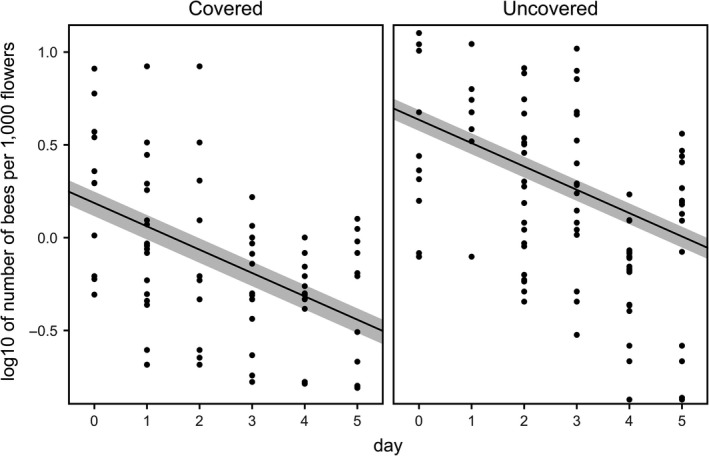
Log‐transformed honeybees observed/1,000 flowers in the focal covered and uncovered kiwifruit orchards throughout the trial. Data are presented as individual quadrant counts (black dots), overall means (black lines), and associated standard errors of the mean (gray area). Means and standard errors are derived from the minimal model. Zeros are not shown due to the data being log‐transformed

Honeybee abundance (bees per 1,000 flowers) in the focal orchards varied with treatment (covered vs. uncovered) and consecutive trial day (Supporting Information Table S3 for model summary). Overall, a greater number of honeybees were observed foraging in the uncovered orchard compared with the covered orchard (2.08 ± 0.25 bees vs. 0.81 ± 0.14 bees per 1,000 flowers; simple effect of treatment significant at *p* < 0.001; LRT: *χ* ^2^ = 16.139). The number of bees per 1,000 flowers decreased in both orchards as the trial progressed (presumably driven by a corresponding increase in the number of flowers); however, the rate of decline was the same across orchards (Figure 3; treatment:day interaction nonsignificant at *p* = 0.67; LRT: *χ* ^2^ = 0.182). Visitation rates (the number of bees visiting focal flowers per hour) also varied with treatment (Supporting Information Table S4 for model summary). Flowers in the uncovered orchard received significantly more visits per hour (5.38 ± 0.61 visits per hour) than those under cover (1.10 ± 0.12 visits per hour; treatment effect significantly different at *p* < 0.001; LRT: *χ* ^2^ = 57.823).

**Figure 4:**
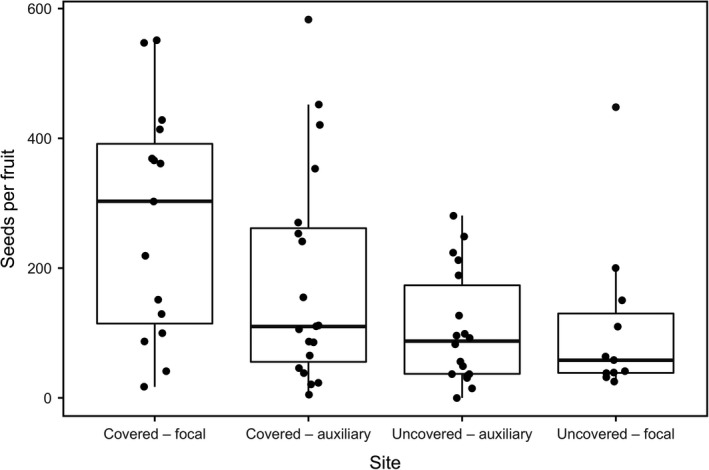
Number seeds produced by kiwifruit flowers that received a single honeybee visit, in covered and uncovered kiwifruit orchards. Overlaid on the raw data, each box indicates the spread between the 25% and 75% percentile, the thick line indicates the median, and the whiskers indicate minimum and maximum values

Honeybee visit duration had no effect on the seed counts from fruit pollinated in a single visit, either in interaction with treatment (GLM; *p* = 0.52) or as a single term (*p* = 0.195). However, the simple effect of the treatment was significant (*p* = 0.012), with counts of seeds in fruit from covered orchards being greater than in fruit from uncovered orchards (Figure 4; Supporting Information Table S5 for model summary). We have little reason to expect that there was pollen movement without insect pollinators, as 97% (35 out of 37) of control (i.e., exposed but unvisited) flowers did not produce a fruit. Two control flowers produced small fruits (31.72 g and 83.68 g, with 18 and 45 seeds, respectively).

Open‐pollinated flowers in both the focal uncovered and covered orchards produced full‐sized, export quality fruit, which contain around 500 seeds (Goodwin et al., 2017). There was a significant interaction between treatment and flower position within orchard (middle vs. edge), with flowers on the edge of the uncovered orchard having fewer seeds (LRT: *χ* ^2^ = 20.803, *p* < 0.001; Supporting Information Table S6 for model summary). However, there was no effect of treatment when position was ignored (LRT: *χ* ^2^ = 3.517, *p* = 0.061), with flowers producing fruit with a similar number of seeds in both the uncovered (506 ± 14 seeds/ fruit) and covered orchards (537 ± 9 seeds/ fruit; Supporting Information Table S7 for model summary). The amount of achieved pollination was therefore similar in both orchards, but in the open orchard pollination was achieved by bees alone and in the covered orchard pollen was applied by both bees and artificial means.

## 4 DISCUSSION

To determine how netting crop covers can affect honeybee foraging dynamics, colony health, and pollination services, we assessed the performance of honeybee colonies and individuals in covered and uncovered kiwifruit orchards. We found that honeybee colonies placed in covered orchards lost adult bees at a faster rate, with colonies losing on average 1,057 ± 274 of their bees in under two weeks. In comparison, colonies in uncovered orchards gained an average of 117 ± 422 bees over the same period. Close observation of individual foragers suggests that the decline in adult bees in the covered orchards was driven by an acute loss of foragers after colonies were moved into the orchard. Under cover, more than 40% of marked bees failed to return to the colony after their first flight; close to three times as many bees as those lost outright from hives not under cover. The remaining bees completed few foraging flights. Both the acute bee loss and behavioral changes could be explained by several factors, including orientation failure and/or “light traps” within the foraging environment.

Honeybees navigate using a combination of the position of the sun in the sky, objective visual landmarks, and the orientation of polarized light when the sun is not visible (Collett, Chittka, & Collett, 2013; Dyer & Gould, 1981; Frisch, 1967). Crop covers are likely to obscure landmarks on the horizon and reduce or alter other visual cues. Hail netting is known to change the quality of sunlight entering growing environments and reduce transmittance of photosynthetically active radiation by between 12% and 25% (Amarante et al., 2011; Middleton & McWaters, 1997). Changes to these navigational cues may be causing failures of navigation and recruitment among foraging honeybees.

The scattering of light by the netting cover may create additional problems for foraging honeybees as it creates relatively bright areas at the ends of kiwifruit rows, or at gaps in the crop canopy. Honeybees are positively phototactic when leaving a foraging source and starting to fly back to their hive (Menzel & Greggers, 1985; Scheiner, Toteva, Reim, Søvik, & Barron, 2014). Bright areas under netting covers may function as “light traps” that attract bees during their return flights. These bees could then become disorientated and lost if they strike the netting or lose sight of relevant landmarks.

Several other factors may explain some of the observed bee losses, but our data suggest that these are not major contributors. Foragers sometimes “drift” between nearby colonies, and this is most likely to occur after their first flight (Free, 1993; Pfeiffer & Crailsheim, 1998). While we did observe drift by our tagged bees into adjacent colonies fitted with readers, the number of occurrences was minimal and higher in the uncovered orchards than under covers (41 and 6 instances, respectively). Similarly, it is unlikely that all the recorded losses were a result of deaths from senescence because foragers of all ages were affected, including older, experienced bees and younger, less experienced bees. Results from control orchards give us confidence that this higher loss of bees under netting covers was very unlikely to have resulted entirely from direct causes of bee mortality (e.g., predation, chilling), or the loss of RFID tags, as these factors would have been similar between treatments.

As well as an acute loss of bees from hives, we observed differences in behavior of successful foragers when under cover, including a lower number and shorter duration of foraging trips, and an increase in short nonforaging flights. This trend was evident among experienced foragers as well as among foraging naïve bees. The short nonforaging trips may have been short‐range orientation flights. Honeybees are observed to make these under adverse foraging conditions including heavy cloud cover, perhaps to reduce the risk of becoming lost (Capaldi et al., 2000; Degen et al., 2015). It is possible that the changes in incident light caused by the covers (see discussion above) may have elicited a similar response from departing bees.

Foragers may have completed shorter foraging trips in the covered orchard because of reduced travel time and flower handling time; they were unable to visit flowers that were located further afield and/or that were morphologically more complex than open kiwifruit flowers, requiring greater handling time (Laverty, 1994). Visual assessment of bees returning to hives in the focal orchards indicated that some bees in the uncovered orchard were collecting pollen from flowers other than kiwifruit, whereas no such foragers were observed in the covered block. It is unlikely that these factors alone were driving the differences in foraging; many foragers in the uncovered orchard were observed collecting kiwifruit pollen. Additionally, during direct observation of bees returning in the covered orchard, very few bees were seen returning with pollen at all, suggesting that few were foraging successfully.

Bee behavior may have been influenced indirectly by environmental changes caused by covers. Protective covers restrict the air flow and radiation reaching the crop underneath, and may alter relative humidity (Gaye, Maurer, & Seywerd, 1991; Loy & Wells, 1975). In addition to the potential to influence flight activity and health (Pinzauti, 1994), these environmental factors could alter floral resource availability of the crop—changing the production of pollen (and nectar in other crops) and in turn eliciting differing responses from foragers (Corbet, 1990; Free, 1993).

Lastly, covers are likely to have follow‐on effects on colony nutrition, as prolonged restriction to a monofloral environment can prevent sustained colony development (Free, 1993). Brood rearing in honeybees requires a sufficient supply of carbohydrates (nectar), water, and a diversity of protein and micronutrients from pollen (Alaux, Ducloz, Crauser, & Conte, 2010; Foley, Fazio, Jensen, & Hughes, 2012; Hendriksma & Shafir, 2016; Di Pasquale et al., 2013). Nutritional limitations may be exacerbated in covered kiwifruit, as kiwifruit provides pollen that is mostly of low (14%) protein content (Clark & Lintas, 1992; Schmid, 1978). The present study does not directly address potential differences in colony demand for resources; this could be achieved by comparing recruitment rates (Gill et al., 2012) or quantifying changes in food stores over time in covered and open environments. Restricted access to nutritional resources could have impacts on brood rearing, limiting the future foraging capacity of the colony even after it is removed from the covered environment. Brood quantity was not assessed in the current study because this invasive procedure is highly disruptive to colony dynamics. However, as brood production directly affects the future foraging strength of a colony, it should be considered in further studies.

Despite the effects on colony strength, and a reduction in bee activity on flowers in covered orchards, we did not detect a corresponding difference in pollination outcomes. It is possible that reduced bee activity was offset by increased pollination efficiency in the covered orchard. Honeybees under covers may exhibit enhanced per‐visit pollination efficiency, as the lack of alternative resources may force them to visit crop flowers more constantly, as opposed to carrying pollen from a variety of plant species (Arceo‐Gómez & Ashman, 2016; Ashman & Arceo‐Gómez, 2013; Morales & Traveset, 2008). However, comparing honeybee contribution to pollination in these different environments is problematic because of low replication, the inherent variability in single visit data (e.g., Howlett, Evans, Pattemore, & Nelson, 2017; Rader et al., 2009), and our inability to precisely control realized stocking rates. Furthermore, milled pollen was applied to kiwifruit flowers in the covered focal orchard. While the pollen was applied at a low rate, it will have contributed directly to the pollination of flowers onto which it was blown (open‐pollinated flowers), and possibly indirectly to all flowers (open‐pollinated and single visit flowers) if it was subsequently moved around by foraging honeybees.

A final consideration regarding pollination outcomes in covered environments is that the stocking rates required for pollination under covers may be quite different from those recommended for open orchards, where honeybee abundance is affected by the temporal variability in the floral resources in the wider landscape. The standard recommendation for uncovered kiwifruit in New Zealand (8–12 hives/ha) is a conservative estimate (Palmer‐Jones et al., 1976). In locations where this represents an overstocking of bees, colonies are likely to increase their foraging range to meet their needs. Using this same stocking rate under covers where bees are confined to kiwifruit flowers may mean there are more bees than necessary for pollination, even with declining numbers of bees in colonies. If the stocking rate in covered environments is too high, this may exacerbate nutrient limitations and colony declines but still provide full pollination for the crop. Separate pollination trials in covered Gold kiwifruit have deployed stocking rates as low as 4 hives/ha without noticeable effects on pollination outcomes (unpublished data, M. Goodwin), and while a conservative stocking rate is still recommended, further research to fine‐tune stocking rates may contribute to more efficient pollination outcomes while reducing pressures on hives. Industry‐wide data on stocking rates and corresponding yields could be useful for refining recommendations; however, Gold3 is a new variety of kiwifruit and has only been grown commercially since 2013 with covers installed shortly thereafter. As such comparative data are not yet readily available.

An acute loss of foragers and changes in the behavior of successful foragers will have implications for ongoing colony health and productivity and may reduce the pollination services provided by each colony. These are important findings as horticultural industries worldwide are increasingly relying on protected cropping for food and fiber production. Enhancing honeybee foraging under nets and increasing the understanding of the effects of nets on bee nutrition and brood rearing should be primary objectives for improving management options for beekeepers and orchard managers.

## CONFLICT OF INTEREST

None declared.

## AUTHOR CONTRIBUTIONS

LJE conceived project. LJE and MG designed the trial. LJE, BTC, MAJ, CF, SC, MJ, and MG conducted the experiment. MJ and LJE analyzed the data. LJE, BTC, MJ, and MAJ wrote the manuscript.

## Data Availability

Data available from the Dryad Digital Repository: https://doi.org/10.5061/dryad.39pt227.
