# Supplementary material for: Netted crop covers reduce honeybee foraging activity and colony strength in a mass flowering crop
Source: Ecol Evol. 2019 Apr 29;9(10):5708–19. doi: 10.1002/ece3.5154 (PMC6540661; doi:10.1002/ece3.5154)
Supplement: Supplementary file 1 [file ECE3-9-5708-s001.docx]

**Title**: Netted crop covers negatively affect honey bee foraging dynamics and hive strength in a mass flowering crop.

Supplementary information

**Authors:** Lisa J. Evans^1*^, Brian T. Cutting^1^, Mateusz Jochym^1^, Milena A. Janke^1^, Crystal Felman^1^, Sarah Cross^1^, Marine Jacob^2^, and Mark Goodwin^1^

^1^Plant & Food Research Australia. c/ Queensland University of Technology, Gardens Point Campus, Brisbane 4001, Australia

^2^Plant & Food Research, Hamilton, 3240, New Zealand

^3^Agrocampus Ouest, 65 Rue de Saint-Brieuc, Rennes 35000, France

* Author for correspondence:

[lisa.evans@plantandfoodresearch.co.nz](mailto:lisa.evans@plantandfoodresearch.co.nz)

**Section S1: Method of estimating number of adult bees in hive**

The number of adult bees present in each hive (a measure of colony strength) was estimated on either the afternoon before or the day the hives were transferred into the orchards. The five frames within each hive were carefully removed one at a time, so as not to disturb the bees, then both sides of the frames were photographed. This process was repeated at the end of flowering (after 9–13days) before removing the colonies from the orchard, to determine whether there had been a change in colony strength. The photos were divided between multiple observers who were blind to the treatment of the hives (located in a covered vs. uncovered orchard) they were assessing. The number of bees present on each frame was approximated by comparing the photograph against a series of 10 ‘standard photographs’ that contained a different known number of bees (150– 1050). Each observer completed both the pre and post assessments for a given hive.

**Section S2: Method of counting seeds per fruit**

The number of seeds inside each fruit was counted by spreading seeds and pulp evenly within a clear plastic bag, then photographing the bag while on top of a tracing light box. These photographs were analysed using the automated object count function within NIS elements software (Nikon), restricting the count to seeds using limitation on object colour wavelengths (RBG), object area, and circularity (the same restrictions were used for all fruit). The seeds from 20 fruit (chosen at random) were also counted by hand to get an estimation of the accuracy of the automated method across all samples. Half of these manual counts were conducted before and half after the automated counts. The automated method counted on average 96 ± 0.9% of the seeds present in each fruit.

**Section S3: Analysis of RFID data**

Raw RFID data were processed with a custom R program (R version 3.3.2; R Core Team 2016). The duration of foraging trips was calculated from the timestamps of RFID tag detections. A worker bee generated the following pattern during a trip cycle: detection at the inner RFID reader followed by detection at the outer RFID reader (‘inner-outer’) indicated a worker leaving the hive; an ‘outer-inner’ pair of detections indicated a returning worker. The duration of a trip was defined by the period between two consecutive detections at the outer reader. The sequences of the trip cycles were not immediately obvious in 65 tagged bees because of a broken pattern of inner-outer-outer-inner detections. Sequences of 12 or more missing detections or isolated pairs of detections (preceded and followed by six or more missing detections) were discarded; in shorter sequences of missing data, the reader pattern was iteratively generated based on the existing reader pattern, i.e. missing ‘outer’ detection was introduced at 0.7 s after preceding existing ‘inner’ detection; missing ‘inner’ detection was introduced at 0.9 s after preceding existing ‘outer’ detection. The delays of 0.7 and 0.9 s (time spent by the bee in transit between outer and inner readers) were based on minimum recorded inbound and outbound transit times of 206 bees with more than two detections and no missing data (i.e. complete reader patterns throughout). Only foraging trips longer than 6 minutes and shorter than 6 hours (started and completed on the same day) were used in the analyses.

**Table S1. Minimal model for colony strength (the number of adult bees present per hive) in the two focal, covered vs. uncovered orchards.**

|  | Model estimate | Standard error | z value | p value |
| --- | --- | --- | --- | --- |
| Intercept | 8.295 | 0.118 | 70.28 | < 0.001 |
| Treatment | 0.174 | 0.167 | 1.04 | 0.30 |
| Day | 0.116 | 0.079 | 1.47 | 0.14 |
| Treatment:Day | -0.216 | 0.106 | -2.02 | 0.04 |

**Summary of the minimal model for colony strength (the number of adult bees present per hive) in the two focal, covered vs. uncovered orchards.**

> Generalized linear mixed model fit by maximum likelihood (Laplace Approximation) ['glmerMod']
> Family: Negative Binomial(48.9794) ( log )
> Formula: count ~ treatment * day + (1 | colony_id)
> Data: dat
>
> AIC BIC logLik deviance df.resid
> 551.6 560.4 -269.8 539.6 26
>
> Scaled residuals:
> Min 1Q Median 3Q Max
> -2.06784 -0.19980 0.02577 0.25907 1.94818
>
> Random effects:
> Groups Name Variance Std.Dev.
> colony_id (Intercept) 0.0906 0.301
> Number of obs: 32, groups: colony_id, 16
>
> Fixed effects:
> Estimate Std. Error z value Pr(>|z|)
> (Intercept) 8.29511 0.11803 70.280 <2e-16 ***
> treatmentcovered 0.17356 0.16685 1.040 0.2982
> day 0.11595 0.07901 1.467 0.1422
> treatmentcovered:day -0.21589 0.10684 -2.021 0.0433 *
> ---
> Signif. codes: 0 '***' 0.001 '**' 0.01 '*' 0.05 '.' 0.1 ' ' 1
>
> Correlation of Fixed Effects:
> (Intr) trtmnt day
> tretmntcvrd -0.707
> day -0.307 0.217
> trtmntcvrd: 0.227 -0.306 -0.740

Fixed effects:

(Intercept) – ‘uncovered’ treatment level

treatmentcovered – ‘covered’ treatment level

day – slope for the consecutive trial day for ‘uncovered’ treatment level

treatmentcovered:day – slope for the consecutive trial day for ‘covered’ treatment level

(interaction term)

Random effects:

colony_id – colony ID

**Table S2. Minimal model for colony strength (the number of adult bees present per hive) in the nine non-focal orchards – four covered and five uncovered.**

|  | Model estimate | Standard error | z value | p value |
| --- | --- | --- | --- | --- |
| Intercept | 8.92 | 0.10 | 87.00 | < 0.001 |
| Treatment | -0.04 | 0.15 | -0.26 | 0.8 |
| Day | -0.02 | 0.11 | -0.15 | 0.88 |
| Treatment:Day | -0.34 | 0.15 | -2.18 | 0.03 |

**Summary of the minimal model for colony strength (the number of adult bees present per hive) in the nine non-focal orchards – four covered and five uncovered.**

> Generalized linear mixed model fit by maximum likelihood (Laplace Approximation) ['glmerMod']
> Family: Negative Binomial(34.3403) ( log )
> Formula: count ~ treatment * day + (1 | colony_id)
> Data: dat
>
> AIC BIC logLik deviance df.resid
> 647.6 657.1 -317.8 635.6 30
>
> Scaled residuals:
> Min 1Q Median 3Q Max
> -1.79517 -0.62646 -0.08284 0.58363 1.64345
>
> Random effects:
> Groups Name Variance Std.Dev.
> colony_id (Intercept) 0.0494 0.2223
> Number of obs: 36, groups: colony_id, 18
>
> Fixed effects:
> Estimate Std. Error z value Pr(>|z|)
> (Intercept) 8.91911 0.10252 86.999 <2e-16 ***
> treatmentcovered -0.03870 0.14994 -0.258 0.7963
> day -0.01576 0.10560 -0.149 0.8814
> treatmentcovered:day -0.33565 0.15433 -2.175 0.0296 *
> ---
> Signif. codes: 0 '***' 0.001 '**' 0.01 '*' 0.05 '.' 0.1 ' ' 1
>
> Correlation of Fixed Effects:
> (Intr) trtmnt day
> tretmntcvrd -0.684
> day -0.625 0.428
> trtmntcvrd: 0.428 -0.598 -0.685

Fixed effects:

(Intercept) – ‘uncovered’ treatment level

treatmentcovered – ‘covered’ treatment level

day – slope for the consecutive trial day for ‘uncovered’ treatment level

treatmentcovered:day – slope for the consecutive trial day for ‘covered’ treatment level

(interaction term)

Random effects:

colony_id – colony ID

**Table S3**. **Minimal model for honey bee abundance; bees per 1000 flowers.**

|  | Model estimate | Standard error | t value | p value |
| --- | --- | --- | --- | --- |
| Intercept | 1.1 | 0.47 | 2.33 | < 0.001 |
| Treatment | -1.08 | 0.24 | -4.60 | < 0.001 |
| Consecutive day of the trial | -0.29 | 0.05 | -6.26 | < 0.001 |

**Summary of the minimal model for honey bee abundance; bees per 1000 flowers.**

> Generalized linear mixed model fit by maximum likelihood (Laplace Approximation) ['glmerMod']
> Family: gaussian ( log )
> Formula: (1000 * bees/n_flowers_per_quadrat) ~ treatment + day_int + (1 | quad) + (1 | time)
> Data: dat
>
> AIC BIC logLik deviance df.resid
> 982.3 1003.0 -485.1 970.3 229
>
> Scaled residuals:
> Min 1Q Median 3Q Max
> -2.6628 -0.3710 -0.1103 0.1387 4.3176
>
> Random effects:
> Groups Name Variance Std.Dev.
> quad (Intercept) 0.4898 0.6999
> time (Intercept) 2.0089 1.4173
> Residual 2.6763 1.6359
> Number of obs: 235, groups: quad, 20; time, 4
>
> Fixed effects:
> Estimate Std. Error t value Pr(>|z|)
> (Intercept) 1.09643 0.47085 2.329 0.0199 *
> treatmentcovered -1.08336 0.23567 -4.597 4.29e-06 ***
> day_int -0.28951 0.04624 -6.262 3.81e-10 ***
> ---
> Signif. codes: 0 '***' 0.001 '**' 0.01 '*' 0.05 '.' 0.1 ' ' 1
>
> Correlation of Fixed Effects:
> (Intr) trtmnt
> tretmntcvrd -0.199
> day_int -0.092 0.223

Fixed effects:

(Intercept) – ‘uncovered’ treatment level

treatmentcovered – ‘covered’ treatment level

day_int – consecutive day of the trial

Random effects:

quad – quadrat

time – time of day

**Table S4. Minimal model for honey bee visitation rates; number of bees visiting focal flowers per hour.**

|  | Model estimate | Standard error | z value | p value |
| --- | --- | --- | --- | --- |
| Intercept | 3.76 | 0.14 | 27.56 | < 0.001 |
| Treatment | -1.58 | 0.18 | -8.84 | < 0.001 |

**Summary of the minimal model for honey bee visitation rates; number of bees visiting focal flowers per hour.**

> Call:
> MASS::glm.nb(formula = (exp(dat$count) - 1) ~ treatment, data = dat,
> init.theta = 1.589847376, link = log)
>
> Deviance Residuals:
> Min 1Q Median 3Q Max
> -3.2565 -0.9008 -0.1132 0.4530 1.4876
>
> Coefficients:
> Estimate Std. Error z value Pr(>|z|)
> (Intercept) 3.7625 0.1365 27.562 <2e-16 ***
> treatmentcovered -1.5837 0.1792 -8.835 <2e-16 ***
> ---
> Signif. codes: 0 '***' 0.001 '**' 0.01 '*' 0.05 '.' 0.1 ' ' 1
>
> (Dispersion parameter for Negative Binomial(1.5898) family taken to be 1)
>
> Null deviance: 180.43 on 89 degrees of freedom
> Residual deviance: 100.63 on 88 degrees of freedom
> AIC: 688.31
>
> Number of Fisher Scoring iterations: 1
>
>
> Theta: 1.590
> Std. Err.: 0.262
>
> 2 x log-likelihood: -682.313

Model terms:

(Intercept) – ‘uncovered’ treatment level

treatmentcovered – ‘covered’ treatment level

**Table S5. Minimal model for number of seeds in single-visit pollinated fruit.**

|  | Model estimate | Standard error | t value | p value |
| --- | --- | --- | --- | --- |
| Intercept | 4.68 | 0.253 | 18.470 | < 0.001 |
| Treatment | 0.72 | 0.278 | 2.596 | 0.0118 |

**Summary of the minimal model for the number of seeds in single-visit pollinated fruit.**

> Call:
> glm(formula = seeds_per_visit ~ treatment, family = gaussian(link = "log"),
> data = dat, mustart = pmax(seeds_per_visit, 0.001))
>
> Deviance Residuals:
> Min 1Q Median 3Q Max
> -215.88 -99.95 -49.45 98.55 362.12
>
> Coefficients:
> Estimate Std. Error t value Pr(>|t|)
> (Intercept) 4.6770 0.2532 18.470 <2e-16 ***
> treatmentcovered 0.7206 0.2776 2.596 0.0118 *
> ---
> Signif. codes: 0 '***' 0.001 '**' 0.01 '*' 0.05 '.' 0.1 ' ' 1
>
> (Dispersion parameter for gaussian family taken to be 21468.93)
>
> Null deviance: 1510988 on 62 degrees of freedom
> Residual deviance: 1309605 on 61 degrees of freedom
> AIC: 811.14
>
> Number of Fisher Scoring iterations: 6

Model terms:

(Intercept) – ‘uncovered’ treatment level

treatmentcovered – ‘covered’ treatment level

**Table S6. Minimal model for number of seeds in open pollinated fruit.**

|  | Model estimate | Standard error | z value | p value |
| --- | --- | --- | --- | --- |
| Intercept | 6.135 | 0.029 | 213.597 | < 0.001 |
| Treatment | 0.186 | 0.038 | 4.839 | < 0.001 |
| Position | 0.180 | 0.041 | 4.394 | < 0.001 |
| Treatment:Position | -0.254 | 0.055 | -4.635 | < 0.001 |

**Summary of the minimal model for number of seeds in open pollinated fruit.**

> Call:
> MASS::glm.nb(formula = n_seeds ~ treatment * pos, data = dat %>%
> na.omit, init.theta = 69.75321907, link = log)
>
> Deviance Residuals:
> Min 1Q Median 3Q Max
> -1.89149 -0.66373 -0.00562 0.53380 2.58820
>
> Coefficients:
> Estimate Std. Error z value Pr(>|z|)
> (Intercept) 6.13535 0.02872 213.597 < 2e-16 ***
> treatmentcovered 0.18556 0.03835 4.839 0.00000130 ***
> posmiddle 0.17982 0.04092 4.394 0.00001113 ***
> treatmentcovered:posmiddle -0.25382 0.05476 -4.635 0.00000356 ***
> ---
> Signif. codes: 0 '***' 0.001 '**' 0.01 '*' 0.05 '.' 0.1 ' ' 1
>
> (Dispersion parameter for Negative Binomial(69.7532) family taken to be 1)
>
> Null deviance: 116.250 on 87 degrees of freedom
> Residual deviance: 88.261 on 84 degrees of freedom
> AIC: 997.73
>
> Number of Fisher Scoring iterations: 1
>
>
> Theta: 69.8
> Std. Err.: 11.9
>
> 2 x log-likelihood: -987.729

Model terms:

(Intercept) – ‘uncovered’ treatment level; position: ‘edge’

treatmentcovered – ‘covered’ treatment level; position: ‘edge’

posmiddle – ‘uncovered’ treatment level; position: ‘middle’

treatmentcovered:posmiddle – ‘covered’ treatment level; position: ‘middle’ (interaction term)

**Table S7. Model for number of seeds in open pollinated fruit, ignoring the position of fruit within the orchards****; ‘Treatment’ term is not significant at p = 0.061 (likelihood ratio tes**t**: 𝜒^2^ = 3.517)**

|  | Model estimate | Standard error | z value | p value |
| --- | --- | --- | --- | --- |
| Intercept | 6.23 | 0.023 | 270.780 | < 0.001 |
| Treatment | 0.06 | 0.031 | 1.896 | 0.058 |

**Summary of the model for number of seeds in open pollinated fruit, ignoring the position of fruit within the orchards; ‘Treatment’ term is not significant at p = 0.061 (likelihood ratio tes**t**: 𝜒^2^ = 3.517)**

> Call:
> MASS::glm.nb(formula = n_seeds ~ treatment, data = dat %>% na.omit,
> init.theta = 53.6213171, link = log)
>
> Deviance Residuals:
> Min 1Q Median 3Q Max
> -2.26686 -0.69727 0.02702 0.54781 2.98806
>
> Coefficients:
> Estimate Std. Error z value Pr(>|z|)
> (Intercept) 6.22699 0.02300 270.780 <2e-16 ***
> treatmentcovered 0.05836 0.03078 1.896 0.058 .
> ---
> Signif. codes: 0 '***' 0.001 '**' 0.01 '*' 0.05 '.' 0.1 ' ' 1
>
> (Dispersion parameter for Negative Binomial(53.6213) family taken to be 1)
>
> Null deviance: 91.914 on 87 degrees of freedom
> Residual deviance: 88.326 on 86 degrees of freedom
> AIC: 1014.5
>
> Number of Fisher Scoring iterations: 1
>
>
> Theta: 53.62
> Std. Err.: 8.90
>
> 2 x log-likelihood: -1008.532

Model terms:

(Intercept) – ‘uncovered’ treatment level

treatmentcovered – ‘covered’ treatment level


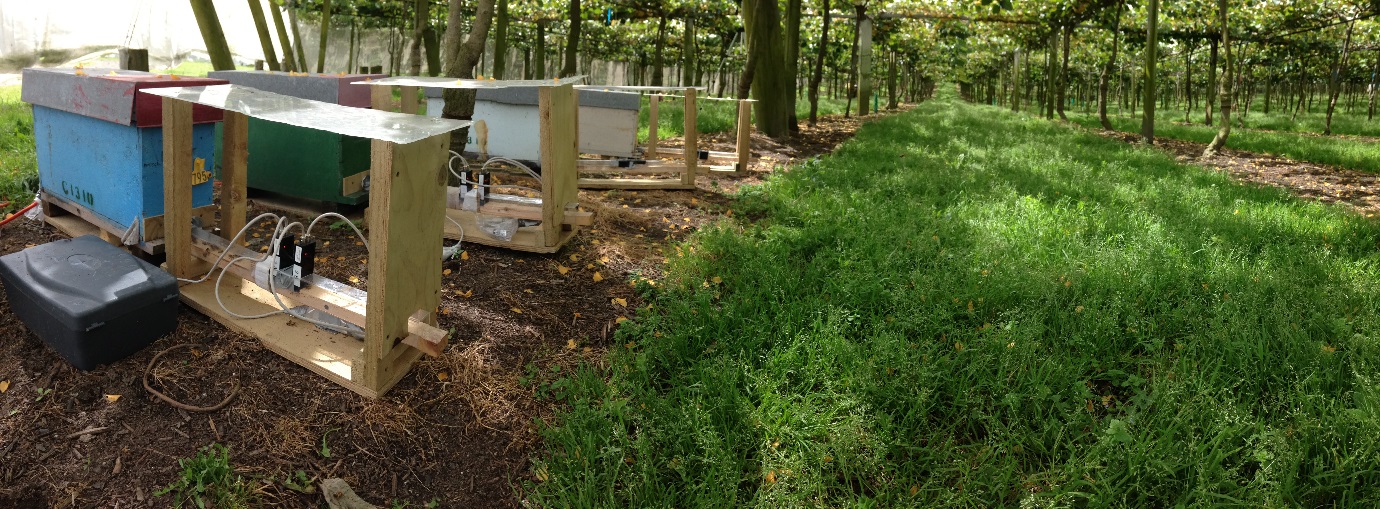


A


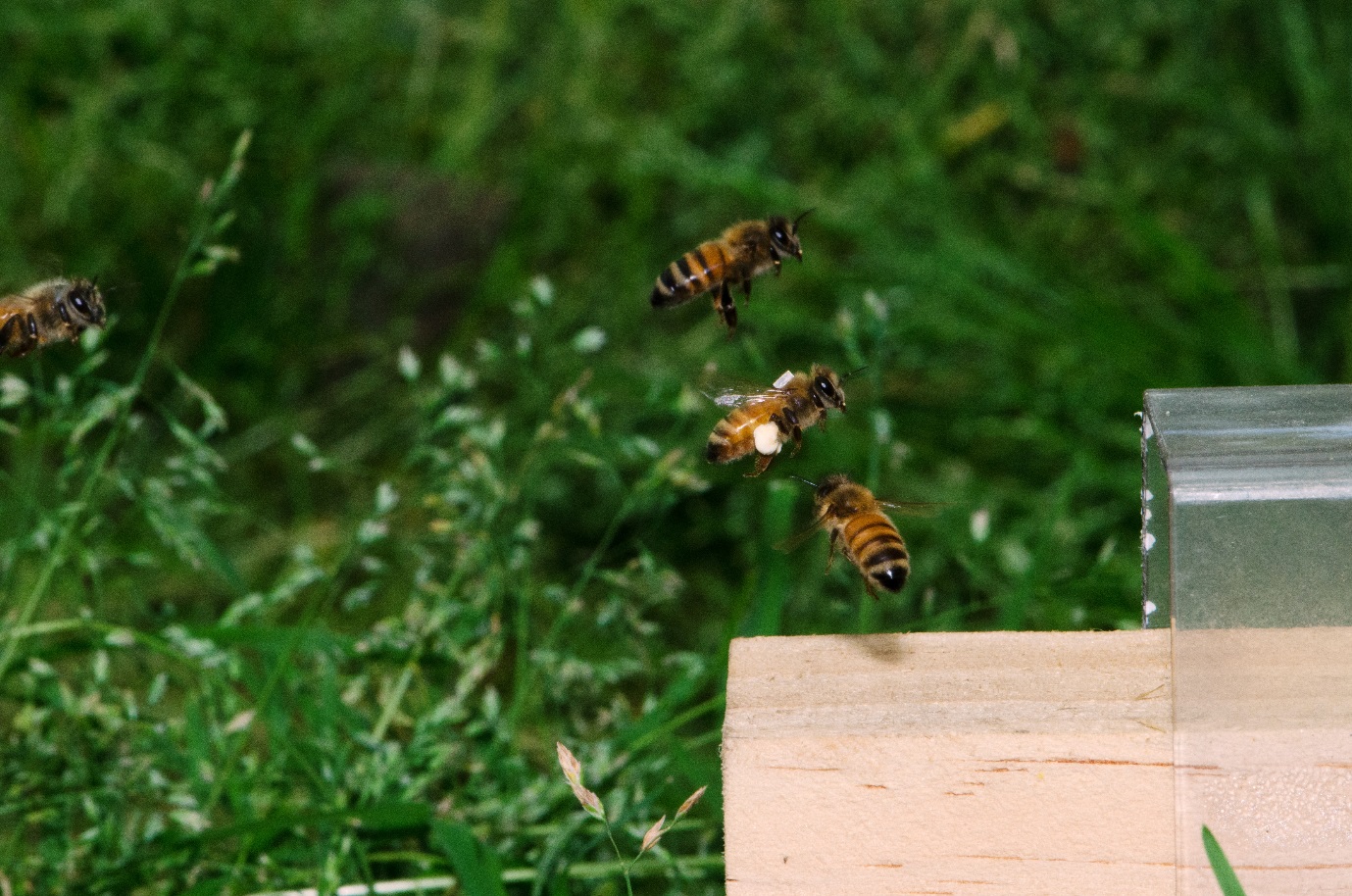


B


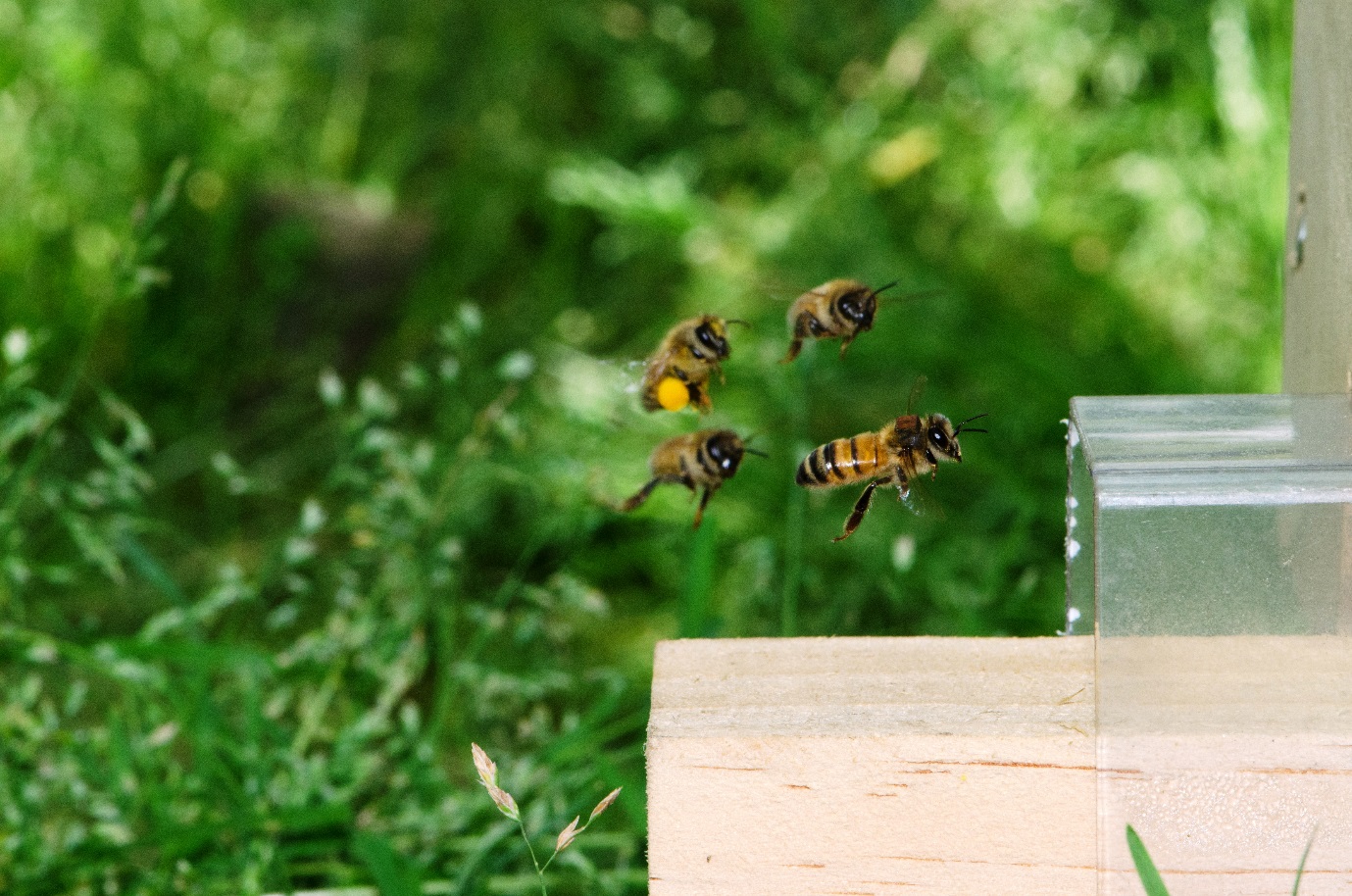


C

**Figure S1.** (A) Observation hives fitted with a modified entrance/exit tunnel (580 x 100 mm) which were transparent to allow direct observation of bees, and allowed fitting of two radio frequency identification (RFID) readers (ilD® MAJA) used to record bi-directional movement of tagged bees in the tunnel. RFID-tagged pollen (B) and nectar (C) foragers (indicated with arrow) returning to their hive after a foraging trip. The tagged pollen forager is carrying cream-coloured kiwifruit pollen in its corbiculae.


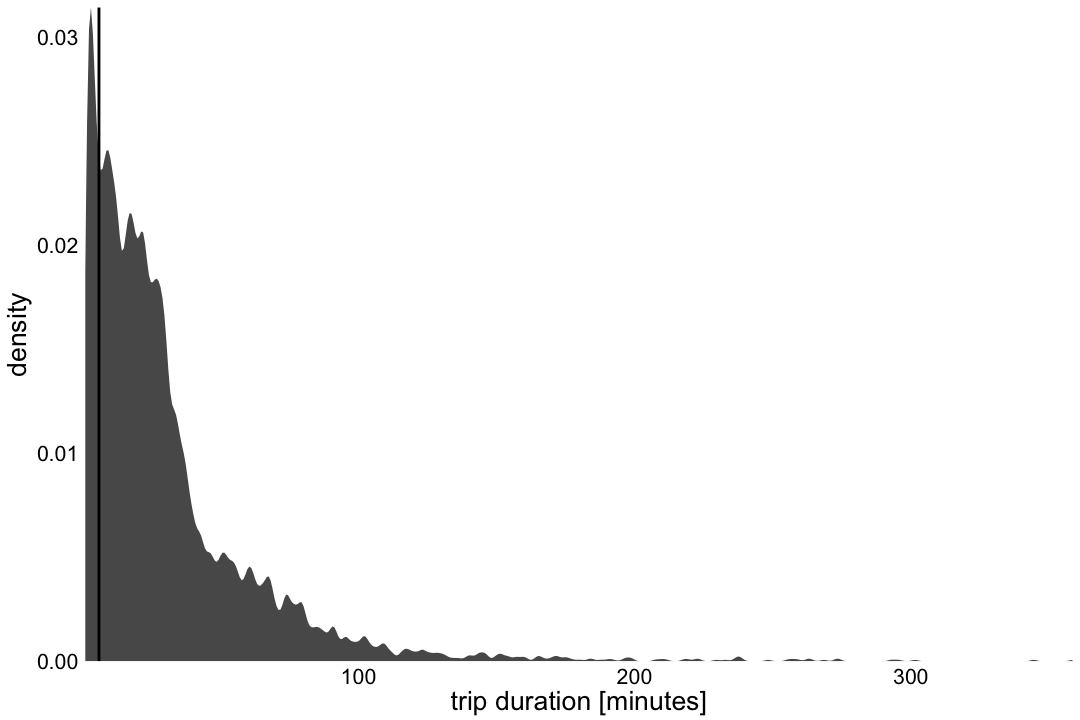

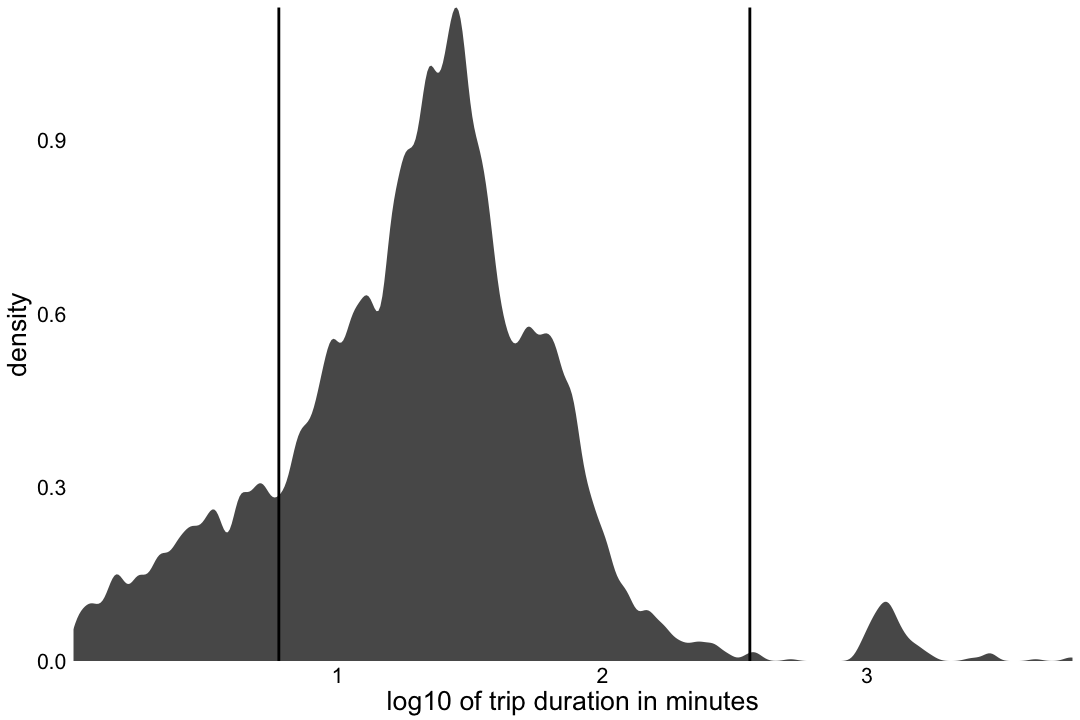


B

A

**Figure S2.** The percentage of the total observed trips to occur within the given trip durations in minutes (A) and log10-transformed minutes (B). Trip duration was defined by the period between two consecutive detections at the outer RFID reader. The two horizontal lines shown in B separate three distinct types of foraging trips: short trips < 6 minutes (far left), foraging trips (centre) and overnight trips > 360 minutes (far right).

A

B

**Figure S3.** Mean number of foraging trips (A) and duration of all individual foraging trips (B) made by different cohorts of RFID tagged bees; foraging naïve (n = 190, 33), nectar (n = 51, 3) and pollen (n = 37, 0) foragers, from eight colonies in a uncovered and eight colonies in a netting covered kiwifruit orchard.
